# Outcomes following repair of incarcerated and strangulated ventral hernias with or without synthetic mesh

**DOI:** 10.1186/s13017-017-0143-4

**Published:** 2017-07-18

**Authors:** Sameh Hany Emile, Hesham Elgendy, Ahmad Sakr, Waleed Ahmed Gado, Ahmed Aly Abdelmawla, Mahmoud Abdelnaby, Alaa Magdy

**Affiliations:** General surgery department, faculty of Medicine, Mansoura University Hospitals, Elgomhuoria Street, Mansoura city, Egypt

**Keywords:** Mesh, synthetic, Ventral hernia, Incarcerated, Strangulated, Outcome, infection

## Abstract

**Background:**

The use of synthetic mesh for ventral hernia repair under contaminated conditions is a controversial issue due to the considerable risk of surgical site infection (SSI). This study aimed to review the outcomes of repair of incarcerated and strangulated ventral hernias with or without synthetic mesh in compliance with established clinical guidelines regarding the incidence of SSI and hernia recurrence.

**Methods:**

The records of patients with complicated ventral hernias who were treated with or without synthetic mesh repair were reviewed. Variables collected included the characteristics of patients and of ventral hernias, type of repair, and incidence of SSI and recurrence.

**Results:**

One hundred twenty-two patients (56 males) of a mean age of 56 years were included. Fifty-two (42.6%) and 70 (57.4%) patients presented with incarcerated and strangulated ventral hernias, respectively. Sixty-six (54%) patients were treated with on-lay mesh repair, and 56 (46%) were managed with suture repair. Twenty-one patients required bowel resection. SSI was detected in eight (6.5%) patients. There was no significant difference between both groups regarding the incidence of SSI (7.5% for mesh group vs 5.3% for suture group). Recurrence occurred in seven patients. Median follow-up period was 24 months. The suture repair group had a significantly higher incidence of recurrence than the mesh group. Diabetes mellitus, previous recurrence, and intestinal resection were significant predictors for SSI.

**Conclusion:**

Following established guidelines, synthetic mesh repair of incarcerated and strangulated ventral hernias attained lower recurrence rate, comparable incidence of SSI, and higher rate of seroma formation than suture repair.

**Trial registration:**

Research Registry, researchregistry1891

## Background

Ventral hernias include all hernias arising from a defect in the anterior abdominal wall as epigastric, umbilical, and incisional hernias. Ventral hernias are susceptible for various complications, particularly incarceration and strangulation, that warrant urgent operative intervention which is usually associated with high rates of postoperative recurrence and complications [1].

The diagnosis of incarceration/strangulation on the verdict of clinical examination alone is not an easy task. Strangulation should be recognized in a timely manner as it warrants immediate surgical intervention. Early operative intervention for strangulated hernia is crucial since any delay in the diagnosis can lead to further compromise of the bowel viability and eventually bowel resection. In addition to the risk of anastomotic leak after bowel resection and anastomosis, strangulation increases the contamination of the operative field which subsequently increases the incidence of postoperative surgical site infection (SSI) and hernia recurrence [2].

The World Society for Emergency Surgery (WSES) [2] has issued a grade 1A recommendation for the use of synthetic mesh repair in the cases of incarcerated ventral hernia without signs of bowel strangulation or concurrent intestinal resection [3, 4]. On the other hand, owing to the increased likelihood of SSI in the cases of strangulated hernias with concomitant bowel resection [5, 6], synthetic mesh repair should be used with caution in these circumstances (grade 2C recommendation).

The use of prosthetic mesh in potentially-contaminated and contaminated settings is not frequently described in the literature. Despite the wide variations of data and the conflicting reports, prosthetic materials are not generally recommended for the repair of ventral hernias in contaminated settings. Choi and colleagues [7] used the records of the National Surgical Quality Improvement Program of the United States and evaluated the prosthetic repair of strangulated ventral hernia for more than 33,000 patients in clean-contaminated and contaminated settings. The authors reported that clean-contaminated cases had a significantly higher incidence of wound disruption, and superficial, and deep SSIs along with significantly increased odds for complications overall.

Xourafas et al. [8] evaluated the feasibility of using a synthetic mesh in ventral hernia repair with simultaneous bowel resection. The authors found a significantly higher incidence of postoperative SSI in patients treated with prosthetic mesh compared to those treated without mesh (22 vs 5%).

The present study aimed to review the outcomes following the repair of incarcerated and strangulated ventral hernias with or without synthetic mesh in compliance with the established clinical guidelines of WSES in terms of the incidence of SSI and recurrence of hernia postoperatively.

## Methods

### Study design and setting

Prospectively collected data of consecutive patients with incarcerated or strangulated ventral hernia who were treated with or without synthetic mesh were reviewed. The study took place at the general surgery departments of Mansoura University principal hospital and Emergency hospital. Data collection involved patients treated between January 2014 and June 2016.

Ethical approval for the study was obtained from the institutional review board of Mansoura Faculty of medicine. The study was registered on www.researchregistry.com under the unique identifying number (UIN): *researchregistry1891.*

### Eligibility criteria

Adult patients of both genders who presented with incarcerated or strangulated ventral hernia were included. Ventral hernias comprised all hernias arising from a defect in the anterior abdominal wall including umbilical, epigastric, incisional, and Spigelian hernias. Patients who underwent resection of gangrenous bowel and anastomosis were also included.

We excluded patients with other types of hernia other than ventral hernias, patients who underwent elective hernia repair after manual reduction of irreducible hernia, patients who underwent bowel resection with stoma formation, patients with incarcerated or strangulated omentum only without bowel involvement, and patients with associated necrotizing fasciitis of the anterior abdominal wall. Patients with incomplete records were also excluded.

### Definition of procedures and outcomes

Incarcerated ventral hernia was defined as irreducible hernia associated with symptoms of bowel obstruction, yet with no compromise of the blood supply of the bowel. Strangulated hernia presented with partial or complete interruption of the blood supply of the intestine. After application of hot fomentation to the doubtful bowel loop, it was considered either viable and returned to the peritoneal cavity or non-viable and resection with anastomosis of the bowel ends was conducted.

Incisional hernias were defined as hernias occurring at the site of abdominal incision performed for procedures other than hernia repair. Recurrent hernias are the hernias occurring at the site of incision made for previous hernia repair. According to the Center for Disease Control and Prevention (CDC) surgical wound classification [9], incarcerated hernias were considered as class I (clean), strangulated hernias without bowel resection as class II (clean contaminated), and strangulated hernias with simultaneous bowel resection as class III (contaminated).

Anesthetic fitness of patients, especially those with associated comorbidities (diabetes mellitus, hepatic disease, hypertension, etc.), was classified according to the American Society of Anesthesiologists (ASA) score into class I to class V. Perioperative antibiotics (2 g of third generation cephalosporines) were administered to patients in both groups in accordance with Surgical Care Improvement Project protocol. The bio-burden reduction technique was followed by careful debridement of all non-viable tissues and removal of mesh in the cases of complicated recurrent hernias after prior mesh repair. The surgical field was thoroughly irrigated with gentamycin-saline solution. Upon completion of repair, subcutaneous drains were routinely placed above the mesh in all patients per hospital protocol and were removed when their drainage was less than 30 cc per day.

Prosthetic mesh repair was performed by approximation of the edges of the abdominal wall defect using continuous running polyprolene 0 sutures then the abdominal wall was reinforced by using a synthetic polyprolene 15 × 15 cm or 30 × 30 cm mesh (Prolene® mesh, Ethicon, Johnson-Johnson Inc. Langhorne, PA, USA) according to the size of the defect. The on-lay technique of mesh repair was used in all patients. Non-mesh suture repair was performed either by direct approximation of the edges of the abdominal wall defect (simple primary repair) or by overlapping the abdominal wall fascia (Mayo’s repair).

The decision on the technique of repair was made by the attending staff surgeon in accordance with the WSES guidelines [2] and depending on the degree of contamination of the surgical field and the general condition of the patients. Patients were primarily offered mesh repair unless they were hemodynamically unstable or had significant comorbidities (i.e., ASA III or higher) warranting a quick intervention such as suture repair. In addition, if patients had significant gross contamination of the surgical field (i.e., spillage of intestinal content, CDC class III) rendering the application of synthetic mesh risky, then suture repair was performed.

Follow-up was conducted at the outpatient clinic by a surgical resident to eliminate risk of investigator bias, and then by one of the operating consultants at 1 week, two weeks, one, six, and 12 months postoperatively then every six months after the first year. Surgical site occurrence (SSO) was defined as SSI, cellulitis, necrosis, chronic and/or non-healing wound, serous or purulent drainage, seroma, hematoma, wound dehiscence, or fistula at the surgical site [10]. SSI was defined according to the standard criteria devised by CDC [9]. Recurrence was defined as the re-appearance of the ventral hernia at the site of previous repair as detected by clinical examination during follow-up.

### Data collection

The records of the patients were searched for the following data: patients’ characteristics as age, sex, ASA grade, and associated comorbidities, type of ventral hernia and type of emergent presentation (incarceration or strangulation), operation performed and whether intestinal resection was done or not, operation time, incidence of SSO, SSI, and recurrence of hernia. The primary outcome of the study was the incidence of postoperative SSI. Secondary end points included the incidence of hernia recurrence after the procedure, SSO, and other postoperative complications.

### Statistical analysis

Excel program and SPSS (Statistical Package for Social Science) version 21 under Microsoft Windows were used to analyze the data. The data was described in the form of mean ± standard deviation (SD) for quantitative data, while frequency and proportion were used to describe qualitative data. Student *t* test was used to compare the quantitative data of the two groups. Fisher’s exact test and Chi square test were used for the analysis of qualitative variables. Multivariate analysis of the risk factors for SSI was performed using the binary logistic regression test. *P* value less than 0.05 was considered significant. Post-hoc analysis of the incidence of recurrence of ventral hernia in both groups (0 vs 12.5%) revealed a study power of 83% with alpha set at 0.05.

## Results

### Patients’ characteristics

Among 145 patients with complicated ventral hernias who were treated within the study period, 122 patients fit the eligibility criteria and were included. Patients were 56 (46%) males and 66 (54%) females. The mean age of patients was 56 ± 10.3 (range, 21–82) years. Ninety-seven (79.5%) patients had associated comorbid conditions: 30 (24.6%) had diabetes mellitus (D.M), 33 (27%) had chronic liver disease (CLD), and 34 (27.8%) had cardiac problems. Eleven (9%) patients had more than one comorbidity. Ninety-nine (81.1%) patients had ASA grade of I-II, whereas 23 (18.9%) patients had ASA grade III-IV. Both groups had comparable demographic data (Table 1).

**Table 1:** Characteristics of patients in both groups

Variable	Mesh repair	Suture repair	Total	*P* value
Number	66	56	122	–
Mean age ± SD (range)	54.6 ± 10.2 (33–82)	57.6 ± 10.4 (21–80)	–	0.11
Male/female	34/32	22/34	56/66	0.24
ASA grade I–II (%)	54 (81.8)	45 (80.3)	99 (81.1)	0.83
ASA grade III–IV (%)	12 (18.1)	11 (19.6)	23 (18.9)	0.83
Diabetes mellitus (%)	16 (24.2)	14 (25)	30 (24.6)	0.92
Hepatic disease (%)	19 (28.7)	14 (25)	33 (27)	0.68
Cardiac disease (%)	19 (28.7)	15 (26.7)	34 (27.8)	0.96
More than 1 comorbidity (%)	7 (10.6)	4 (7.1)	11 (9)	0.54

### Characteristics of ventral hernia

One hundred and three (84.4%) hernias were umbilical, six (5%) were epigastric, ten (8.1%) were incisional, and three (2.4%) were Spigelian. Thirteen patients (10.6%) had a recurrent umbilical hernia after previous operations (four previous mesh repairs and nine suture repairs). The median size of hernia defect was 7 (range, 5–14) cm.

Fifty-two (42.6%) and 70 (57.4%) patients presented with incarcerated and strangulated ventral hernias, respectively. No significant differences were noted between the two groups regarding the characteristics of ventral hernias as illustrated in Table 2.

**Table 2:** Characteristics of ventral hernia in both groups

Variable		Mesh repair (*n* = 66)	Suture repair (*n* = 56)	Total (*n* = 122)	*P* value
Primary/recurrent hernia		58/8	51/5	109/13	0.78
Anatomic location	Umbilical (%)	57 (86.3)	46 (82.1)	103 (84.4)	0.69
	Epigastric (%)	4 (6)	2 (3.57)	6 (5)	0.68
	Spigelian (%)	1 (1.5)	2 (3.57)	3 (2.4)	0.59
	Incisional (%)	4 (6)	6 (10.7)	10 (8.1)	0.51
Incarcerated hernia (%)		31 (47)	21 (37.5)	52 (42.6)	0.38
Strangulated hernia (%)		35 (53)	35 (62.5)	70 (57.4)	0.38
CDC wound classification	Class I	31 (47)	21 (37.5)	52 (42.6)	*0.00003*
	Class II	33 (50)	16 (28.5)	49 (40.1)	
	Class III	2 (3)	19 (34)	21 (17.2)	

*CDC* center for disease control

The italicized figures indicate a signficant *p*-value less than 0.05

### Treatment and outcomes 

**Fig. 1:**
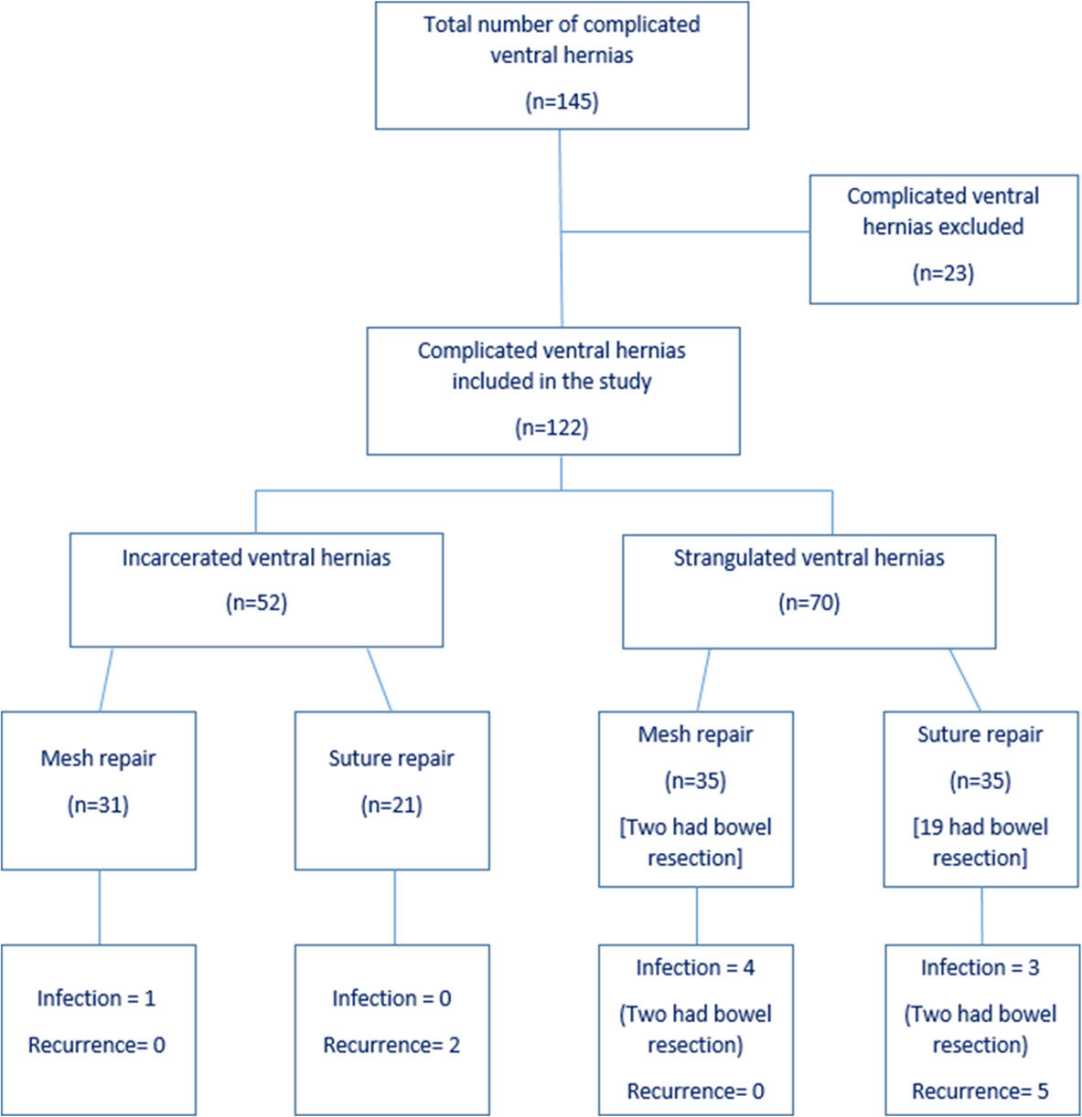
Flow diagram illustarting the treatment and outcomes of patients with complicated ventral hernias

Sixty-six (54%) patients were treated with prosthetic mesh repair, and 56 (46%) were managed with suture repair (simple primary repair in 39 and Mayo’s repair in 17 patients). Mesh repair was done in 31 (60.7%) of 52 incarcerated hernias and 35 (50%) of 70 strangulated hernias (Table 3, Fig. 1).

**Table 3:** Outcome of surgical treatment in both groups

Variable		Mesh repair (*n* = 66)	Suture repair (*n* = 56)	Total (*n* = 122)	*P* value
Median operation time in minutes (range)		88 (70–120)	68 (46–128)	77 (46–128)	**–**
Resection anastomosis (%)		2 (3)	19 (34)	21 (17.2)	*<0.0001*
SSO (%)	SSI (%)	5 (7.5)	3 (5.3)	8 (6.5)	0.72
	Seroma (%)	15 (22.7)	4 (7.1)	19 (15.5)	*0.02*
	Hematoma (%)	3 (4.5)	1 (1.7)	4 (3.2)	0.62
	Wound dehiscence (%)	1 (1.5)	2 (3.5)	3 (2.4)	0.59
	Total (%)	24 (36.3)	10 (17.8)	34 (27.8)	*0.038*
SSI (%)	In incarcerated hernia	1/31 (3.2)	0/21 (0)	1/52 (1.9)	1
	In strangulated hernia	4/35 (11.5)	3/35 (8.5)	7/70 (10)	1
Recurrence (%)		0	7 (12.5)	7 (5.7)	*0.003*
Median follow-up in months (range)		24 (6–32)	22 (6–30)	–	–

*SSI* surgical site infection, *SSO* surgical site occurrence

The italicized figures indicate a signficant* p*-value less than 0.05

Twenty-one (17.2%) patients underwent resection of gangrenous bowel with anastomosis of the bowel ends. Two (9.5%) of those 21 patients were treated with mesh hernioplasty and 19 (90.5%) were treated with suture repair.

The suture group had a significantly higher rate of contaminated (CDC class III) wounds. Overall, there were 52 (42.6%) class I wounds, 49 (40.1%) class II wounds, and 21 (17.2%) class III wounds. Contents of the incarcerated and strangulated hernia comprised small bowel alone (*n* = 88), small bowel and omentum (*n* = 12), large bowel alone (*n* = 8), large bowel and omentum (*n* = 14).

SSO was detected in 34 (27.8%) patients including SSI (*n* = 8), seroma (*n* = 19), hematoma (*n* = 4), and wound dehiscence (*n* = 3). The mesh hernioplasty group had significantly (*p* = 0.038) higher rate of SSO overall, particularly seroma formation, than the suture group.

SSI in the form of superficial incisional infection was detected in eight (6.55%) patients, seven of them presented with strangulated ventral hernia and one with incarcerated hernia, three (5.3%) of which were in the non-mesh group and five (7.5%) were in the mesh group. Patients who developed SSI were treated conservatively on outpatient basis with antibiotics for 7–14 days according to the culture results and drainage if necessary. Only one patient in the mesh group was re-admitted for 3 days to have his mesh surgically removed owing to resistant SSI unresponsive to conservative treatment. All cases of SSI were detected within 30 days after surgical repair; there were no cases of latent mesh infection on further follow-up for at least 12 months.

There was no significant difference (*p* = 0.72) between both groups regarding the overall incidence of SSI (7.5% [95%CI: 2.9–16.9%] for the mesh group vs 5.3% [95%CI: 1.2–15.1%] for the suture group). Similarly, on subgroup analysis, no significant differences in the rates of SSI were observed between mesh and suture repairs in the cases of incarcerated hernias (*p* = 1) and strangulated hernias (*p* = 1).

Seven (5.7%) patients developed recurrence of ventral hernia within a period ranging from 6–18 months after repair, all recurrences were in the suture repair group. The suture repair group had a significantly (*p* = 0.003) higher incidence of recurrence than the mesh group (Table 3).

The median duration of follow-up was 24 months (range, 6–32 months). Ninety-two (75.4%) patients were followed for more than 18 months, 21 (17.3%) were followed for 12-18 months, and nine (7.3%) were followed for less than 12 months.

### Risk factors for SSI

Univariate analysis (Table 4) of the risk factors for SSI after surgical repair revealed that D.M (*p* = 0.02), previous recurrence (*p* = 0.04), and intestinal resection (*p* = 0.03) were the significant predictors for SSI. All previously recurrent hernias that developed SSI had prior mesh repair. Four of 21 patients who had resection of gangrenous bowel with re-anastomosis developed SSI, two of which had mesh repair and another two had suture repair.

**Table 4:** Univariate and multivariate analysis of the risk factors for SSI

Variable	Infection (*n* = 8)	No infection (*n* = 114)	Univariate analysis	Multivariate analysis		
			*P* value	Odds ratio	95% CI	*P* value
Mean age ± SD	56.2 ± 5.8	56 ± 10.6	0.96	**–**	**–**	**–**
Male patient (%)	3 (37.5)	53 (46.5)	0.72	**–**	**–**	**–**
Female patient (%)	5 (62.5)	61 (53.5)	0.72	**–**	**–**	**–**
Diabetes mellitus (%)	6 (75)	24 (21)	*0.002*	11.2	2.13–59.31	*0.004*
Hepatic disease (%)	3 (37.5)	30 (26.3)	0.44	**–**	**–**	**–**
More than 1 comorbidity (%)	2 (25)	9 (7.9)	0.15	**–**	**–**	**–**
ASA grade III–IV (%)	1 (12.5)	22 (19.3)	1	**–**	**–**	**–**
Previous recurrence (%)	3 (37.5)	10 (8.8)	*0.04*	6.24	1.29–30	*0.02*
Umbilical hernia (%)	7 (87.5)	96 (84.2)	1	**–**	**–**	**–**
Incisional hernia (%)	1 (12.5)	9 (7.9)	0.5	**–**	**–**	**–**
Strangulation (%)	7 (87.5)	63 (55.2)	0.13	**–**	**–**	**–**
CDC class III wound	4 (50)	17 (14.9)	*0.03*	5.7	1.3–25.03	*0.02*
Resection anastomosis (%)	4 (50)	17 (14.9)	*0.03*	5.7	1.3–25.03	*0.02*
Mesh repair	5 (62.5)	61 (53.5)	0.72	**–**	**–**	**–**
Suture repair	3 (37.5)	53 (46.5)	0.72	**–**	**–**	**–**

The italicized figures indicate a signficant* p*-value less than 0.05

Binary logistic regression analysis revealed the most significant risk factors for SSI included D.M (OR = 11.2, *p* = 0.004), previous recurrence (OR = 6.2, *p* = 0.02), and intestinal resection with CDC class III wound (OR = 5.7, *p* = 0.02).

Patients’ age, gender, and ASA grade; type of ventral hernia; type of emergent presentation; and type of repair were not significant risk factors for the development of SSI postoperatively.

## Discussion

The debate on the optimal management of complicated ventral hernias continues. The use of prosthetic mesh has been considered the standard treatment for elective ventral hernia repair since simple repair with sutures is associated with significantly higher rates of recurrence of hernia that can reach up to 67% on long term follow-up [11, 12]. On another hand, the emergency management of complicated ventral hernias can be more cumbersome owing to the potential contamination of the operative field which can preclude the use of synthetic mesh. Alternatives to synthetic mesh, as bioprosthesis and biosynthetic mesh, appeared to solve the problem; nevertheless, these alternatives are not readily available everywhere because of their cost. This leaves the surgeon on the horns of a dilemma that entails higher risk for SSI when synthetic mesh is used and higher incidence of recurrence when the mesh is abandoned.

The WSES [2] has issued a useful set of recommendations for the emergency repair of complicated ventral hernias including a grade A1 recommendation for the use of synthetic mesh for incarcerated hernias, grade 2C recommendation for the use of synthetic mesh with caution in strangulated hernias with or without concurrent bowel resection, and grade 2C recommendation for using biologic mesh instead of synthetic mesh in the cases of acute strangulation with bowel perforation.

We employed the WSES clinical guidelines for emergency ventral hernia repair on 122 patients and examined the outcome of synthetic mesh repair versus suture repair performed in accordance with the guidelines. Patients included were adults of a mean age of 56 years close to the mean age of paraumbilical hernia reported by Bedewi et al. [13]. The majority of the patients had at least one associated comorbidity, yet less than 20% of them had high ASA grade. Umbilical hernias represented around 85% of the ventral hernias included in the study, in accord with Aguirre and colleagues [14] defined this type as the most common type of ventral hernia.

Both groups (suture and mesh repair) had comparable patients’ demographics and hernia characteristics which can minimize the inherent risk of selection bias associated with retrospective studies. Although according to WSES guidelines, incarcerated hernias are recommended to be managed with synthetic mesh repair invariably, only 60% of these patients were treated with mesh repair in our study. The reason for this can be the overall general condition and associated comorbidities of the patients that called for a rapid maneuver such as simple suture repair. On the other hand, only half of the patients with strangulation and around 10% of the patients with concomitant bowel resection underwent mesh repair which is in line with the WSES recommendations to use synthetic mesh cautiously in these potentially contaminated conditions.

Although mesh hernioplasty had higher incidence of SSO overall, there was no significant difference in the incidence of SSI between mesh repair and suture repair. The overall 30-day SSI after mesh repair in our series was 7.5% ranging between 3% for incarcerated hernias and 11.5% for strangulated hernias. The rates of SSI after prosthetic repair of strangulated hernias are generally higher than those of incarcerated hernia which can be attributed to the ischemia of the bowel wall that predisposes to bacterial translocation, as well as the risk of bowel resection that can further increase the contamination of the surgical field. That is why the operative field in the cases of strangulated hernias was classified as clean-contaminated or contaminated [2].

A randomized controlled trial [15] found prosthetic repair of incarcerated umbilical hernias a safe option with lower recurrence rates than suture repair. Similarly, other retrospective studies [16–18] concluded that synthetic mesh repair was not associated with higher risk of SSI in the treatment of complicated ventral and groin hernias. Unlike the present study, the study by Bessa and Abdel-Razek [17] lacked the presence of a control group (suture repair) to compare the outcomes with.

The incidence of SSI in the present series was comparable to that disclosed by Nieuwenhuizen et al. [18], slightly higher than the incidence (6%) reported by Abd Ellatif and colleagues [19], yet much lower than the rate of SSI (14%) Carbonell and colleagues [20] reported after the application of synthetic mesh in contaminated ventral hernia repair. Nonetheless, since causes of contamination in Carbonell’s series were quite variable including infected previous mesh, violation of the gastrointestinal tract, and ostomy creation or reversal along other causes whereas contamination in our series was caused by bowel strangulation only; the higher rate of SSI in their study can be explained. We excluded patients with stoma from the present report since the management of stoma in conjunction with ventral hernia is likely to be coupled with serious morbidities as Carbonell et al. have acknowledged.

The relatively low incidence of SSI after synthetic mesh repair in the current study can be attributed to the combined application of clinical guidelines along with the use of good clinical judgment. As a general rule, following guidelines and applying evidence-based medicine can serve to achieve better outcomes in the surgical practice. The incidence of SSI after prosthetic mesh repair of complicated ventral hernias can be further reduced by employing different techniques of mesh placement (i.e., retrorectus mesh) and negative pressure wound therapy.

Despite the low rates of SSI in our report, chronic infection after the use of synthetic mesh under contaminated conditions remains one of the most concerning consequences. Chronic infection can compromise the wound healing resulting in chronic draining sinuses. Additionally, involvement of the underlying abdominal viscera can sometimes result in enterocutaneous fistulas. Furthermore, re-admission and reoperation for the management of mesh-related infection, including mesh extraction, are linked with added morbidities to the patient and higher treatment-related costs.

Another controversial issue is the feasibility of synthetic mesh repair when bowel resection is being conducted in the same setting. Less than 10% of patients with bowel resection in our study were treated with a synthetic mesh. We found bowel resection and anastomosis to be a significant independent risk factor for SSI whether a mesh was used or not. Four of 21 patients with bowel resection developed SSI, attaining a rate of 19% close to the incidence (22%) reported by Xourafas and colleagues [8] when simultaneous intestinal resection took place with synthetic mesh repair of ventral hernia. Bowel resection with possibility of spillage of intestinal content into the operative field renders the field a class III, contaminated surgical wound. The use of synthetic mesh in such a contaminated setting should be employed with caution as per the WSES guidelines.

Conversely, other investigators found the use of synthetic mesh to be acceptable with bowel resection. Geisler and affiliates [21] examined the safety of non-absorbable mesh repair in the presence of open bowel and reported a wound-related morbidity rate of 7%. Campanelli et al [22] recommended prosthetic repair to be used in all cases of abdominal wall hernia even in potentially contaminated fields; nevertheless, since the authors operated on only ten patients, the outcome of their study should be interpreted with caution. Other studies [17, 19] came to a conclusion that intestinal resection should not be considered a contraindication to the use of synthetic mesh.

In addition to bowel resection, D.M and previous hernia surgery were recognized as significant predictors for SSI. D.M has been known to be associated with higher rates of infection after mesh repair of hernia [23] since it reduces the T cell response and neutrophil function and causes disorders of humoral immunity [24]. Previous surgeries for ventral hernias, especially when involving the use of prosthetic mesh, may render the repeat procedure technically demanding with difficult dissection, longer operation time, and increased risk of inadvertent bowel injury during dissection of the adhesions. Moreover, if the recurrence of hernia was associated with surgical site infection, further procedures for the treatment of recurrent hernia can have higher likelihood of SSI.

Although on-lay mesh hernioplasty was reported to have higher rates of recurrence and SSI than the sub-lay technique [25], we found the on-lay repair confers satisfactory results with no recorded cases of hernia recurrence and a rate of SSI comparable to those recorded after the sub-lay technique which ranged from 0–14.4%. We opt for employing the on-lay technique of repair as it appeared technically easier and faster to perform in the setting of emergency treatment of complicated ventral hernia, unlike the sub-lay technique that can be more technically demanding, time consuming, and requires better experience. Moreover, we thought that if SSI occurred, the removal of on-lay mesh would be technically easier and safer than the removal of a deeply-placed retromuscular mesh. Nevertheless, we think if the mesh was placed in retro-rectus position, better results could have been obtained with lower rates of SSO and SSI.

Reviewing the current literature, two opposite perspectives can be noted; the first discourages the use of mesh repair for ventral hernias at any level of contamination [7], and the second recommends using mesh repair even with evident contamination as in the cases of concomitant bowel resection [17, 19]. Based on the outcomes of the present study, we would choose a compromise between the two extremes. Since prosthetic mesh repair attained significantly lower incidence of recurrence of hernia than suture repair without significantly increasing the rates of SSI, we recommend the use of synthetic mesh hernioplasty for complicated ventral hernias, with exception of diabetic patients, previously recurrent hernias, and when bowel resection is to be simultaneously conducted.

Even with bowel resection, patients should not be denied the option of reinforcing the abdominal wall with mesh to minimize the likelihood of recurrence. Some options were devised in this context as the retro-rectus positioning of the polyprolene mesh. Proper preparation, washing, and accurate drainage of the preperitoneal space in addition to perfect hemostasis and local antibiotic treatment are crucial to guarantee the success of this method [22].

The use of bioprosthesis has been proposed as a safe and efficient approach for abdominal wall reconstruction under contaminated conditions, yet the long term durability of biologic mesh has not been verified [26]. Other alternatives include the use of long-term absorbable synthetic materials or the biosynthetic meshes. The use of biosynthetic (GORE BIO-A) has been evaluated in The Complex Open Bioabsorbable Reconstruction of the Abdominal Wall (COBRA) study [27] which concluded the efficacy of biosynthetic prosthesis in terms of long-term recurrence and life quality with a SSI of around 18%.

Limitations to the present study include being a single-institution, retrospective study, the small number of patients who had bowel resection, and the lack of some essential parameters as the body mass index, cigarette smoking, and steroid use that may have an impact on the incidence of SSI after the treatment of complicated ventral hernias. Furthermore, the technique of prosthetic repair used (on-lay mesh) is not widely used which may limit the generalizability of the study. Finally, the non-randomized nature of the study and the selection of patients for each treatment group according to the established guidelines and clinical judgment may have been the cause of the better outcomes reported, rather than the type of repair per se.

## Conclusion

Following the WSES clinical guidelines in conjunction with good clinical judgment, synthetic mesh repair of incarcerated and strangulated ventral hernias achieved lower recurrence rate than suture repair. Although mesh hernioplasty was followed by a higher incidence of SSO and seroma formation, the incidence of SSI was comparable in both groups.

Bowel resection, D.M, and previously recurrent ventral hernias were the significant independent risk factors for SSI after repair of complicated ventral hernias. Well-powered randomized trials are needed to reach a strong conclusion about the safety of synthetic mesh when simultaneous bowel resection is conducted; until then, synthetic mesh should be used with caution in these conditions.

## Abbreviations

CDC: Center of disease control
CLD: Chronic liver disease
D.M: Diabetes mellitus
OR: Odds ratio
SSI: Surgical site infection
SSO: Surgical site occurrence
